# Synthesis and Structural Characterization of Bioactive PHA and γ-PGA Oligomers for Potential Applications as a Delivery System

**DOI:** 10.3390/ma9050307

**Published:** 2016-04-25

**Authors:** Iwona Kwiecień, Iza Radecka, Michał Kwiecień, Grażyna Adamus

**Affiliations:** 1 Centre of Polymer and Carbon Materials, Polish Academy of Sciences, Zabrze 41-819, Poland; ikwiecien@cmpw-pan.edu.pl (I.K.); mkwiecien@cmpw-pan.edu.pl (M.K.); 2 School of Biology, Chemistry and Forensic Science, Faculty of Science and Engineering, University of Wolverhampton, Wolverhampton WV1 1SB, UK; I.Radecka@wlv.ac.uk

**Keywords:** biodegradable polymers, polyhydroxyalkanoates, P(3HB-*co*-4HB), poly-γ-glutamic, γ-PGA, conjugates, tyrosol, mass spectrometry

## Abstract

The (trans)esterification reaction of bacterial biopolymers with a selected bioactive compound with a hydroxyl group was applied as a convenient method for obtaining conjugates of such compound. Tyrosol, a naturally occurring phenolic compound, was selected as a model of a bioactive compound with a hydroxyl group. Selected biodegradable polyester and polyamide, poly(3-hydroxybutyrate-*co*-4-hydroxybutyrate) (P(3HB-*co*-4HB)) and poly-γ-glutamic acid (γ-PGA), respectively, were used. The (trans)esterification reactions were carried out in melt mediated by 4-toluenesulfonic acid monohydrate. The structures of (trans)esterification products were established at the molecular level with the aid of ESI-MS^2^ (electrospray ionization tandem mass spectrometry) and/or ^1^H NMR (nuclear magnetic resonance) techniques. Performed analyses confirmed that the developed method leads to the formation of conjugates in which bioactive compounds are covalently bonded to biopolymer chains. The amount of covalently bonded bioactive compounds in the resulting conjugates depends on the type of biopolymers applied in synthesis.

## 1. Introduction

Design of biodegradable delivery systems for bioactive substances is a rapidly developing field. The aim of designing delivery systems is modification of properties of bioactive molecules, such as solubility, stability and bioactivity, and improve the delivery efficiency. For these purposes biodegradable, biocompatible and nontoxic polymeric carriers might be applied [1,2,3,4]. Potential uses of polyhydroxyalkanoates (PHAs) in polymeric delivery systems of bioactive compounds have been evaluated in a number of studies—for example, delivery systems for steroids [5], antibiotics [6,7], or antitumor agents [8,9].

Polyhydroxyalkanoates are aliphatic polyesters produced under controlled conditions via biotechnological processes using numerous microorganisms and by varying the producing strains and substrates. The PHAs are accumulated as granules in the cell, reaching 90% levels of dry cell mass [10]. In this way, a number of polyesters which differ in monomer composition have been synthesized [11,12]. It was essential to search for different inexpensive raw materials for PHA production, and it is already known that these biopolyesters can be produced from renewable resources and from a broad range of waste and surplus materials, such as glycerol from biodiesel production, protein hydrolysates, meat and bone meal from slaughtering and rendering industries or molasses from the sugar industry [13,14,15].

Previously, we reported (i) one-pot synthesis in which the respective bioactive compound-oligomer conjugates were obtained through the transesterification reaction of PHA biopolyesters by selected bioactive compounds with a carboxyl group in the presence of 4-toluenesulfonic acid monohydrate and (ii) a two-step procedure of obtaining bioactive PHA conjugates, designed for bioactive compounds with hydroxyl group; in the first step, cyclic oligomers were obtained from bacterial poly(3-hydroxybutyrate) according to the method described in the literature [16]; subsequent lipase-catalyzed transesterification of the cyclic oligomers by the bioactive compounds leads to the formation of the conjugates [17]. The proposed synthetic strategy for bioactive compounds with a hydroxyl group is too complex and economically disadvantageous for industrial application. The method of coupling of specific bioactive compounds (containing a hydroxyl group) to biodegradable polymeric carriers has been presented. The elaborated method is a promising way of obtaining delivery systems for retarded release of bioactive compounds. Our hypothesis was that the (trans)esterification reaction of bacterial biopolymers with a bioactive compound with a hydroxyl group may prove to be a more simple and economically favorable method for obtaining such conjugates.

Tyrosol was selected as a model of bioactive compound with a hydroxyl group. Tyrosol is a naturally occurring phenolic compound found, for example, in olive oil and wine [18,19]. Tyrosol and its derivatives show interesting biological properties, such as antioxidant [20] and anticancer effects [21] as well as preventing inflammation-induced osteopenia [22] and smoking-induced oxidative stress [23].

The tyrosol-polymer conjugates were synthesized via (trans)esterification reaction of tyrosol with selected biopolymers (such as poly(3-hydroxybutyrate-*co*-4-hydroxybutyrate) (P(3HB-*co*-4HB)) or poly-γ-glutamic acid (γ-PGA)) carried out in melt under an argon atmosphere mediated by 4-toluenesulfonic acid monohydrate (TSA·H_2_O). To the best of our knowledge and according to relevant information, there are no earlier reports on the use of the (trans)esterification method for the synthesis of delivery systems of tyrosol based on such biopolymers.

## 2. Results

It was found that the “one-pot” transesterification method developed by us for obtaining conjugates of bioactive compounds with the carboxyl group [17] could also be an implement for preparing conjugates of bioactive compounds with a hydroxyl group.

**Scheme 1:**
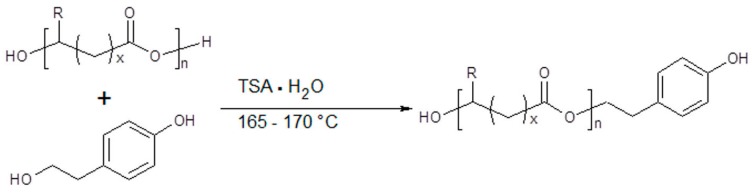
Transesterification reaction of P(3HB-*co*-4HB) by tyrosol, mediated by TSA·H_2_O. R = CH_3_, *x* = 1–3HB units; R = H and *x* = 2–4HB units.

The tyrosol-oligo(3HB-*co*-4HB) conjugates were synthesized via a transesterification reaction of P(3HB-*co*-4HB) with tyrosol in the presence of TSA·H_2_O (20 wt %) carried out in melt under an argon atmosphere (Scheme 1). The results of the experiments performed are summarized in Table 1. The amount of TSA·H_2_O was chosen based on our previous experience, wherein the relationship between the number average molar masses (*M*_n_) of the resulting conjugates and the amount of TSA·H_2_O used in the process was experimentally determined [17]. In our previous research, it was observed that increasing the quantity of water, which was introduced with TSA, led to the decrease of the molar mass of resulting oligomers [17]. The optimal molar masses of the oligomers was obtained by using of 20 wt % of TSA·H_2_O; therefore, the amount of TSA·H_2_O used in the each synthesis in this study was the same and equaled the 20 wt %. From inspection of Table 1, it can be observed that increasing the quantity of bioactive substance led to the decrease of molar mass of resulting products. Moreover, based on these results (shown in Table 1), it was determined that the amount of bioactive substance used in transesterification reactions influenced the dispersity index *M*_w_/*M*_n_. Through increasing the amount of tyrosol, a decrease of dispersity index was achieved.

The structures of obtained conjugates were preliminarily characterized by GPC (gel permeation chromatography) and ^1^H NMR techniques (results summarized in Table 1). Further structural studies were performed with the aid of ESI-MS (electrospray ionization mass spectrometry). Tyrosol-oligo(3HB-*co*-4HB) conjugates with number average molar masses in the range 850–1800 g/mol were obtained.

**Figure 1:**
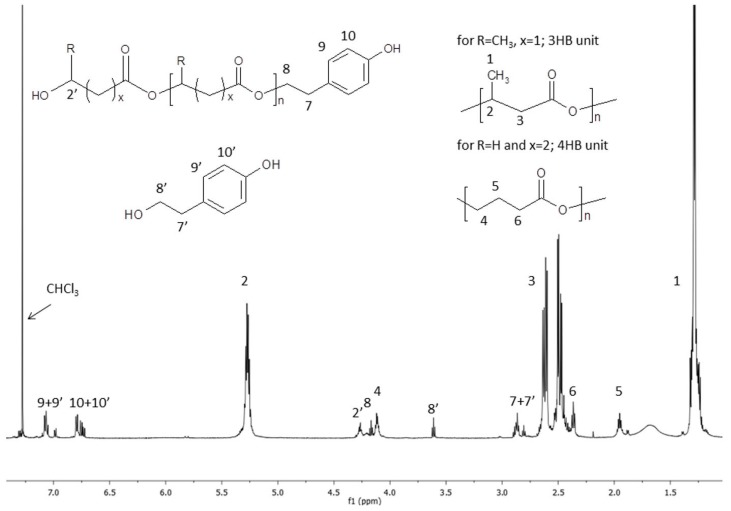
The ^1^H NMR spectrum of Sample 2 from Table 1.

The ^1^H NMR spectrum of the products obtained through the transesterification reaction of P(3HB-*co*-4HB) with tyrosol mediated by TSA·H_2_O (Sample 2, Table 1) is presented in Figure 1. In this spectrum, signals corresponding to the protons of both comonomeric units were observed, signals labelled 1–3 correspond to the protons of the 3-hydroxybutyrate repeating units and signals labeled 4–5 correspond to the protons of the 4-hydroxybutyrate repeating units. The signals corresponding to the protons of the tyrosol molecule bonded to the oligomers were labeled 7–10, while signals of the protons of the unbonded tyrosol molecule were labelled 7’–10’.

In order to obtain more detailed structural information about the obtained conjugates, the ESI-MS^n^ technique was applied. Recently, ESI-MS has been successfully applied in the structural studies of conjugates of biodegradable oligomers and bioactive substances, such as food preservatives [24], herbicides [25,26], non-steroidal anti-inflammatory drugs [27,28], or antioxidants used in cosmetology [29,30,31].

**Figure 2:**
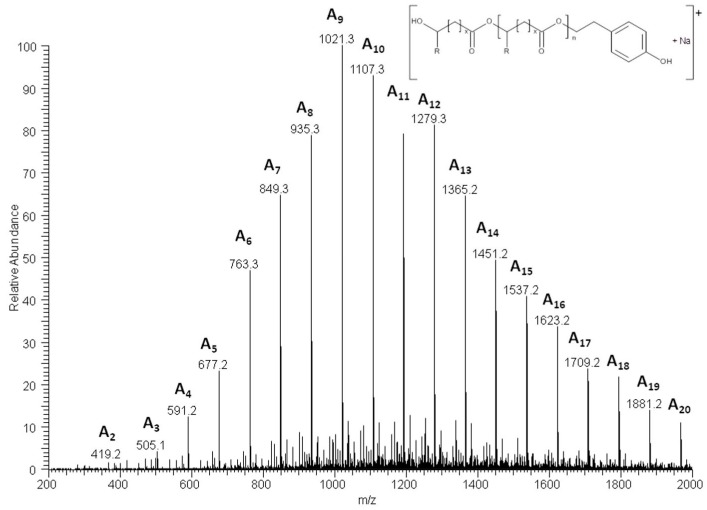
ESI-MS spectrum in positive-ion mode of the tyrosol-oligo(3HB-*co*-4HB) conjugates obtained by transesterification reaction between P(3HB-*co*-4HB) and tyrosol (for 3HB units R = CH_3_, *x* = 1, for 4HB units R = H and *x* = 2).

The ESI-MS spectrum (in positive-ion mode) of the selected tyrosol-oligo(3HB-*co*-4HB) conjugate (Sample 3, Table 1) is presented in Figure 2. The spectrum consists of singly charged ions. The main series of ions correspond to sodium adduct of tyrosol-oligo(3HB-*co*-4HB) conjugates with tyrosol and hydroxyl end groups. The additional series of signals (however, with significant lower intensity) visible on the spectrum correspond to: sodium adduct of oligomers with hydroxyl and carboxyl end groups (which formed due to the partial hydrolysis of biopolyesters), oligomers terminated by crotonate and carboxyl end groups (formed due to the partial thermal degradation of biopolyesters), as well as oligomers terminated by crotonate and tyrosol end groups which are the result of transesterification between tyrosol and oligomers formed due to the partial thermal degradation of biopolyesters.

In order to further structural clarification of tyrosol-oligo(3HB-*co*-4HB) bioconjugate structure assignment (series A, Figure 2), tandem mass spectrometry (ESI-MS^2^) was used.

**Figure 3:**
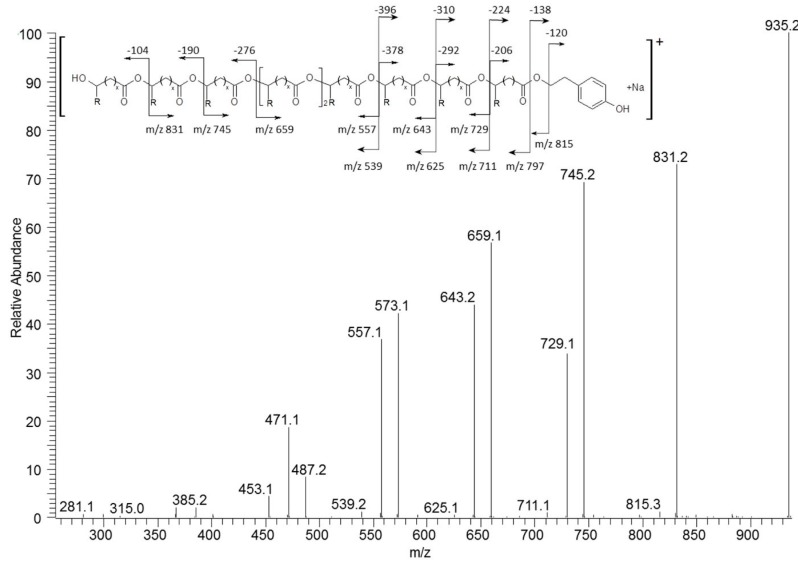
ESI-MS/MS product ion spectrum of the sodiated tyrosol-oligo(3HB-*co*-4HB) conjugate at *m*/*z* 935 and theoretical fragmentation pathway of this ion.

Figure 3 presents ESI-MS^2^ spectrum (in positive-ion mode) of the precursor ion at *m*/*z* 935 corresponds to sodium adduct of tyrosol-oligo(3HB-*co*-4HB). Fragmentation of this ion, which proceeds via β-hydrogen rearrangement at the ester groups, leads to the random breakage of ester bonds along the 3HB-*co*-4HB oligomer chain accompanied by an expulsion of neutral molecules and to the creation of product ions) (see scheme of Figure 3). According to the structures assigned the product ion at *m*/*z* 831 corresponds to the oligomer formed by the loss of 3-hydroxybutyric acid (104 Da), the product ion at *m*/*z* 815 corresponds to the oligomer formed by the loss of 4-ethenylphenol (120 Da), the product ion at *m*/*z* 797 corresponds to the oligomer formed by the loss of tyrosol (138 Da) and the product ion at *m*/*z* 729 corresponds to the oligomer formed by the loss of 2-(4-hydroxyphenyl)ethyl crotonate (206 Da)(see scheme on Figure 3).

The conjugates of tyrosol and bacterial poly(3-hydroxybutyrate-*co*-4-hydroxybutyrate) described above contain one bioactive molecule per oligomer chain. However, conjugate of bioactive compounds with an increased amount of biologically active moieties along the oligomer chain seems to be a much more interesting option. For the synthesis of such conjugates of poly-γ-glutamic acid (γ-PGA), polymers made of d- or l-glutamic acid units connected by amide linkages [32], were used as carriers. The γ-PGA is naturally occurring polymer that is also biodegradable, edible and non-toxic toward humans and the environment, produced extracellularly by several species of bacteria of the genus *Bacillus* classified as GRAS (Generally Regarded As Safe) by the US Food and Drug Administration [33,34]. Poly-γ-glutamic acid has been already tested as a carrier for bioactive substances, for example paclitaxel [35], cisplatin [36], insulin [37] and heparin [38].

**Scheme 2:**
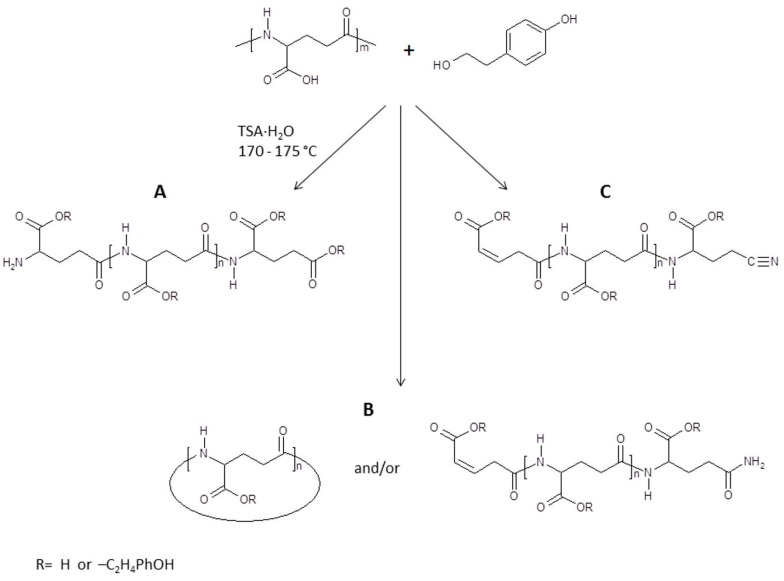
Esterification reaction of γ-PGA by tyrosol, mediated by TSA·H_2_O; (**A**) oligomers with amino and carboxyl end groups; (**B**) cyclic amides and/or oligomers with unsaturated alkyl and amide end groups; (**C**) oligomers with unsaturated alkyl and nitriles end groups.

For preparing conjugates of tyrosol and γ-PGA, similar procedures as for conjugates of tyrosol-oligo(3HB-*co*-4HB) were applied. Esterification reactions were carried out in melt in the presence of TSA·H_2_O (20 wt %) (Scheme 2). Under these conditions, carboxyl groups along the γ-PGA chain should undergo esterification with tyrosol molecules while peptide bonds along the polymer chain could be hydrolyzed due to the presence of the water introduced.

It is known that, as a result of thermal degradation of polyamides, various products could be obtained. Heating of polypeptides in the presence of water, leads to hydrolysis of peptide bonds [39]. The *cis*-eliminations are also known mechanisms of thermal decomposition of polyamides through a 6-membered ring which causes NH-CH_2_ bond scission and leads to oligomers with unsaturated alkyl and amide end groups further transformed into nitriles [40,41,42]. Other known products of thermal degradation are cyclic amides, which are result of intramolecular amide exchange [41,42,43].

Based on our previous results as well as other literature information we predicted that esterification reaction of γ-PGA with tyrosol, which is carried out in melt, can lead to oligomer with different structures. In order to obtain detailed structural information about obtained products, *i.e.*, the structures of the end groups and amount of repeating units in each conjugate oligomers, the ESI-MS technique was used.

**Figure 4:**
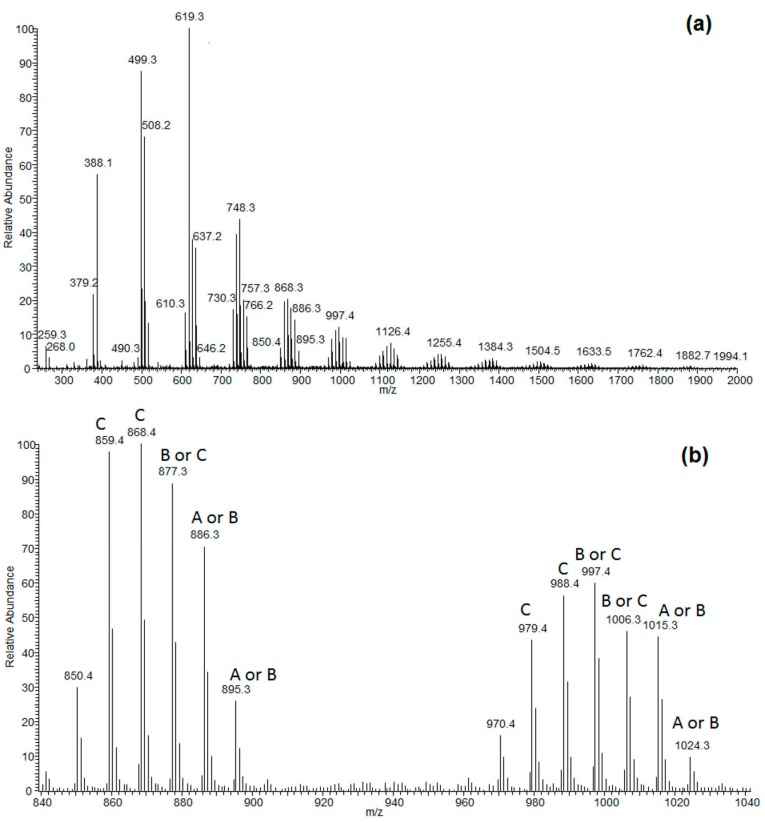

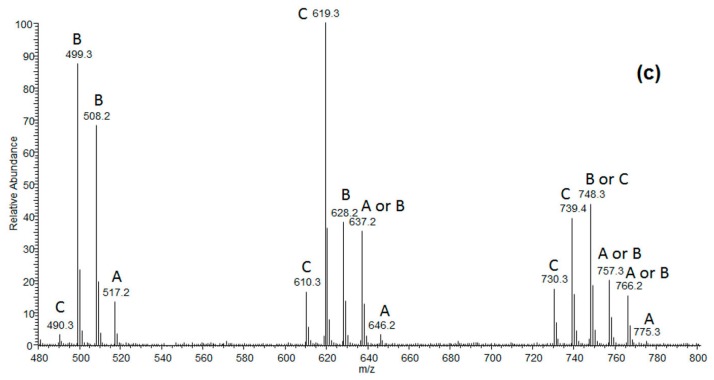
ESI-MS spectrum (in positive-ion mode) of the tyrosol-γ-PGA conjugates obtained by esterification reaction between γ-PGA and tyrosol (**a**) and spectral expansion in the range *m*/*z* 840–1040 (**b**) and *m*/*z* 480–800 (**c**).

In Figure 4, ESI-MS spectrum of the product obtained in reaction between tyrosol and γ-PGA in the presence of TSA·H_2_O is presented.

The presence of a tyrosole molecule in the obtained conjugate can be expected at each degree of polymerization and the amount of this bioactive substance increases with an increase in the degree of polymerization. For example, γ-PGA dimers can be bonded with a maximum of three bioactive molecules due to presence of three carboxyl groups, while γ-PGA tetramer can be bonded for up to five bioactive molecules due to the presence of five carboxyl groups.

From the inspection of mass spectrum (Figure 4), it can be observed that a higher degree of polymerization increases the number of possible structures of tyrosol-γ-PGA conjugates (see Figure 4b,c).

The structures of the ions visible on the mass spectrum, which represent the γ-PGA oligomers obtained in esterification carried out in melt, were assigned based on different mechanisms of thermal decomposition of polyamides discussed in literature [40,41,42,43,44]. The proposed structures were placed in Table 2.

**Figure 5:**
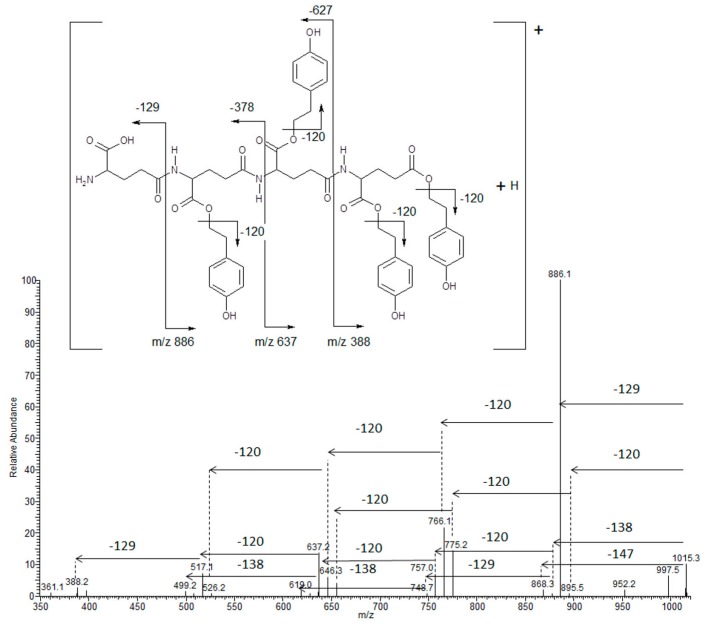
ESI-MS/MS product ion spectrum of the sodiated tyrosol-γ-PGA conjugate at *m*/*z* 1015 and theoretical fragmentation pathway of this ion.

In order to establish the structure of tyrosol-γ-PGA conjugates, ESI-MS^2^ analyses were performed for selected molecules containing different amounts of bioactive molecule per oligomer chain. Figure 5 shows ESI-MS^2^ spectra of conjugates containing four bioactive molecules per oligomer chain.

Figure 5 presents ESI-MS^2^ spectrum (in positive-ion mode) of the precursor ion at *m*/*z* 1015 corresponding to γ-PGA oligomers containing four tyrosol molecules per one oligomer chain. One of the possible structures of ion at *m*/*z* 1015 is shown on the scheme in Figure 5. The product ion at *m*/*z* 997 corresponds to the oligomer formed by the loss of the water (18 Da). The product ion at *m*/*z* 895 corresponds to the oligomer formed by the loss of the 4-ethenylphenol (120 Da). The product ion at *m*/*z* 886 corresponds to the oligomer formed by the loss of the pyroglutamic acid, 5-membered lactam (129 Da), from the N-terminal end. The product ion at *m*/*z* 877 corresponds to the oligomer formed by the loss of the tyrosol (138 Da). The product ion at *m*/*z* 868 corresponds to the oligomer formed by the loss of the γ-glutamic acid, 147 Da, (in cases when the last repeating unit is not esterified) from C-terminal end.

The structural verification of the ions at *m*/*z* 1015 corresponding to tyrosol-γ-PGA oligomer conjugates with the aid of ESI-MS^2^ techniques confirmed that those oligomers contained four tyrosole units, which are covalently bonded to γ-PGA chains. Moreover, esterified carboxyl groups are randomly distributed along the obtained conjugate chains.

## 3. Discussion

Previously, the two-step method for preparing conjugates of bioactive compound with a hydroxyl group was reported by some of us. In the first step, cyclic oligomers of PHB (poly(3-hydroxybutyrate)) were obtained; in the second step, these cyclic oligomers were applied in transesterification reactions with bioactive compounds in the presence of enzymes. The novel application of synthetic strategies described here based on (trans)esterification of biopolymers with bioactive compounds with a hydroxyl group turned out to be a relatively quick, simple and solvent-free way of obtaining conjugates of biopolymers with such bioactive compounds.

In this study, P(3HB-*co*-4HB) biopolymer was used to obtain conjugates with tyrosol via transesterification reaction mediated by TSA·H_2_O. Similarly, as was previously established, increasing the quantity of water, which is introduced with TSA to the reaction medium, led to the decrease of molar mass of resulting oligomers [17]. Moreover, based on results shown in Table 1, it was determined that amount of bioactive substance used in transesterification reactions influenced the dispersity index *M*_w_/*M*_n_. Through increasing the of amount of tyrosol, a decrease of dispersity index was achieved.

The transesterification of P(3HB-*co*-4HB) biopolyester with tyrosol leads to conjugates which contain one bioactive molecule per oligomer chain. The poly-γ-glutamic acid is a polyamide which contains carboxyl groups in each repeating unit, and these carboxyl groups allow for coupling bioactive moieties to each repeating unit. Therefore, application of the γ-PGA, as a polymeric carrier provides a possibility to increase the amount of biologically active moieties along the oligomer chain.

Applying γ-PGA in our study was successful and allowed us to obtain conjugates of bioactive compounds with the γ-PGA oligomer chain. As was expected, the conjugates we synthesized contained a higher amount of biologically active moieties distributed along the γ-PGA oligomer chain. The amount of tyrosol molecules which are bonded to the γ-PGA oligomer chain increased with an increase of molar masses of γ-PGA oligomers. For example, γ-PGA dimer can be bonded with a maximum of three bioactive molecules due to the possibility of esterification by tyrosol of the three carboxyl groups present in this oligomer, while γ-PGA tetramer can be bonded up to five bioactive molecules due to presence of five carboxyl groups.

The tyrosol-γ-PGA conjugates were obtained in an esterification reaction between tyrosol and γ-PGA biopolymer carried out in melt. Under these reaction conditions, the esterification between tyrosol and γ-PGA biopolymer can be accompanied by thermal decomposition of γ-PGA, which is typical for polyamides. The presence of tyrosol-γ-PGA conjugates with different structures in the products of esterification reaction between tyrosol and γ-PGA was identified using mass spectrometry. However, it is noteworthy that all types of obtained γ-PGA oligomers contain bioactive molecules bonded along oligomer chains, which was confirmed using electrospray ionization tandem mass spectrometry.

The structural characterization of the tyrosol-γ-PGA with the aid of ESI-MS^2^ techniques allowed the structure of those conjugates at the molecular level to be established. Moreover, these studies confirmed that esterified by tyrosol carboxyl groups are randomly distributed along γ-PGA conjugate chains.

Determination of the most appropriate molar mass of tyrosol-oligo(3HB-*co*-4HB) and tyrosol-γ-PGA conjugates, as well as the influence of the presence of various structures of the end groups in tyrosol-γ-PGA conjugates on properties and potential applications of this conjugates as a delivery system of tyrosol, will be investigated in further studies.

## 4. Materials and Methods

### 4.1. Materials

The poly(3-hydroxybutyrate-*co*-4-hydroxybutyrate) was purchased from Tianjin Green Bio-Science (Tianjin, China); the number-average molar mass, as determined by GPC, was *M*_n_ = 250,000 g/mol and the dispersity index was *M*_w_/*M*_n_ = 2.5. The 4HB unit content was 8.8 mol % (based on the ^**1**^H NMR spectrum). The poly-γ-glutamic acid was purchased from Natto Biosciences (Montreal, QC, Canada), *M*_n_ = 47,800 g/mol, *M*_w_/*M*_n_ = 3.2. Additionally, chloroform, hexane and *N*,*N*-Dimethylformamide (DMF) were supplied by POCH SA (Gliwice, Poland). The 4-(2-hydroxyethyl)phenol (tyrosol) and 4-toluenesulfonic acid monohydrate were purchased from Sigma-Aldrich Chemie GmbH (Steinheim, Germany). Dialysis membrane Spectra/Por (MWCO (molecular weight cut-off): 12,000–14,000) was purchased from Carl Roth (Karlsruhe, Germany).

### 4.2. Measurements

Gel permeation chromatography (GPC) analyses were carried out using Viscotek VE 1122 pump (Malvern Instruments Ltd., Worcestershire, UK), Shodex SE-61 RI detector (JM Science Inc., Grand Island, New York, NY, USA), and PLgel 3 μm MIXED-E (Polymer Laboratories, Santa Clara, CA, USA) high-efficiency column (300 mm × 7.5 mm). Analyses were performed at 35 °C using CHCl_3_ as mobile phase with the 1 mL/min flow rate. The instrument was calibrated with polystyrene narrow standards.

Nuclear magnetic resonance (NMR) analyses were performed using NMR Spectrometer Avance II 600 MHz Ultrashield Plus (Bruker, Rheinstetten, Germany); CDCl_3_ was used as the solvent and tetramethylsilane was used as the internal standard.

Electrospray mass spectrometry (ESI-MS^n^) analyses were performed in positive-ion mode using a Thermo LCQ Fleet ion-trap mass spectrometer (Thermo Fisher Scientific Inc., San Jose, CA, USA). Solutions of samples were introduced into the ESI source by continuous infusion at a 10 μL/min flow rate using the instrument syringe pump. Settings and conditions: spray voltage: 5.0 kV, capillary temperature: 200 °C, sheath gas: nitrogen, auxiliary gas: helium. For ESI-MS/MS experiments, precursor ion was isolated by the ion trap and collisionally activated.

### 4.3. Synthesis of Conjugates

Biopolymer (0.25 g) and an appropriate amount of bioactive substance (amount of tyrosol for synthesis of PHA-based conjugates is placed in Table 1; for synthesis of PGA-based conjugates 0.1 g of tyrosol was used) and 4-toluenesulfonic acid monohydrate (TSA·H_2_O) (0.05 g; 20 wt % compared to biopolymer) were placed into a round bottom flask equipped with a magnetic stirring bar. The vessel, containing reagents, was placed into a Heat-On Block System (RB Radley & Co. Ltd., Essex, UK) located on a stirring hot plate. Reactions were carried out in the melt (at 167–172 °C) under an argon atmosphere. The molten reagents were stirred for 90 s; then, the reaction mixture was cooled. The PHA-based conjugates were purified by added 5 cm^3^ of chloroform and washed 5 times with distilled water to remove any residual 4-toluenesulfonic acid. Then, the products were precipitated with cold hexane and dried under a vacuum at room temperature. The PGA-based conjugates were dissolved in DMF; solutions were dialyzed through a dialysis tubes for 72 h, precipitates settled at the bottom of the dialysis bag. Precipitated products were dried under a vacuum at room temperature. The yield of reactions was between 60% and 78%.

## 5. Conclusions

The method for the preparation of conjugates consisting of bioactive compounds with hydroxyl group (tyrosol) and oligomers from bacterial biopolymers has been discussed in detail. The developed method of (trans)esterification of selected biopolymers (such as P(3HB-*co*-4HB) or γ-PGA) in the presence of tyrosol mediated by 4-toluenesulfonic acid monohydrate is occurring in melt. This one-pot synthesis method is rapid and does not use solvents, and, therefore, is promising from a scale-up perspective because of the relatively low cost of reagents and the rather simple procedure.

The structural characterization at the molecular level of the trans-esterification products with the aid of ESI-MS^2^ and/or ^1^H NMR techniques confirmed that the developed method leads to the formation of conjugates in which bioactive compounds are covalently bonded to biopolymer chains.

It was shown that transesterification of P(3HB-*co*-4HB) with tyrosol leads to the (3HB-*co*-4HB) oligomers that contain one bioactive molecule covalently bonded to the oligomer chain, while esterification of γ-PGA with tyrosol results in conjugates with increased amount of biologically active moieties along the oligomer chain. Thus, unexpectedly, it was found that esterification reactions of γ-PGA with tyrosol carried out in melt in the presence of TSA·H_2_O enabled the preparation of conjugates containing from one to seven bioactive molecule per oligomer chain, which was confirmed using electrospray ionization tandem mass spectrometry. The signals in ESI mass spectrum correspond to individual macromolecular ion of conjugates; therefore, based on the mass assignment of singly charged ions observed in the mass spectrum, we were able to established amount of repeating units in each conjugate oligomers (degree of oligomerization) as well as amount of bioactive molecules per oligomer chain.

The developed biodegradable polymeric systems should allow for the delivery of tyrosol, thereby prolonging and improving the efficacy of this bioactive compound. The release of tyrosol should be accompanied by the formation of non-toxic degradation products of P(3HB-*co*-4HB) or γ-PGA carriers.

## Figures and Tables

**Table 1:** Results of the transesterification reaction of P(3-HB-*co*-4HB) by tyrosol in the presence of 4-toluenesulfonic acid monohydrate (TSA·H_2_O) (20 wt %).

Sample	Tyrosol (wt %)	*M*_n_ (g/mol)	*M*_w_/*M*_n_
1	10	1800	3.16
2	20	1600	2.58
3	30	1000	2.11
4	40	850	1.87
5	50	950	1.76

The amount of tyrosol compared to the amount of biopolymer (0.25 g) used for the transesterification reactions, expressed in weight percent. The *M*_n_ and *M*_w_/*M*_n_ values were determined using GPC (gel permeation chromatography). The *M*_n_ and *M*_w_/*M*_n_ values for P(3-HB-*co*-4HB)biopolymer were equal *M*_n_ = 250,000 g/mol; *M*_w_/*M*_n_ = 2.5.

**Table 2:** Structural assignments of the most intensive ions appearing in the expanded regions at *m*/*z* 480–800 and 840–1040 of the ESI–MS spectrum.

Structure		Ions (*m*/*z*)
A	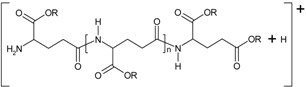	517; 637; 646; 757; 766; 775; 886; 895; 1015; 1024; 1135; 1144; 1153
B	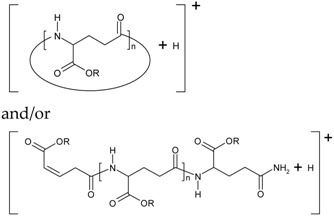	499; 508; 628; 637; 748; 757; 766; 877; 886; 895; 997; 1006; 1015; 1024; 1126; 1135; 1144; 1153
C	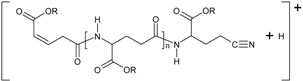	490; 610; 619; 739; 748; 859; 868; 877; 979; 988; 997; 1006; 1108; 1117; 1126

R = H or –C_2_H_4_PhOH.
